# Complement Activation Is Associated With Mortality in Patients With Necrotizing Soft-Tissue Infections—A Prospective Observational Study

**DOI:** 10.3389/fimmu.2020.00017

**Published:** 2020-01-31

**Authors:** Markus Korsholm Kristensen, Marco Bo Hansen, Martin Bruun Madsen, Cecilie Bo Hansen, Katrine Pilely, Ole Hyldegaard, Peter Garred

**Affiliations:** ^1^Laboratory of Molecular Medicine, Department of Clinical Immunology, Rigshospitalet, University Hospital of Copenhagen, Copenhagen, Denmark; ^2^Department of Anesthesia, Center of Head and Orthopedics, Rigshospitalet, University Hospital of Copenhagen, Copenhagen, Denmark; ^3^Department of Intensive Care, Rigshospitalet, University Hospital of Copenhagen, Copenhagen, Denmark

**Keywords:** necrotizing fasciitis, soft tissue infection, sepsis, amputation, survival complement activation, immune system

## Abstract

**Aim:** We assessed whether different complement factors and complement activation products were associated with poor outcome in patients with necrotizing soft-tissue infection (NSTI).

**Methods:** We conducted a prospective, observational study in an intensive care unit where treatment of NSTI is centralized at a national level. In 135 NSTI patients and 65 control patients, admission levels of MASP-1, MASP-2, MASP-3, C4, C3, complement activation products C4c, C3bc, and terminal complement complex (TCC) were assessed.

**Results:** The 90-day mortality was 23%. In a Cox regression model adjusted for sex, and SAPS II, a higher than median MASP-1 (HR 0.378, CI 95% [0.164–0.872], *p* = 0.0226) and C4 (HR 0.162, 95% CI [0.060–0.438], *p* = 0.0003), C4c/C4 ratio (HR 2.290 95% CI [1.078–4.867], *p* = 0.0312), C3bc (HR 2.664 95% CI [1.195–5.938], *p* = 0.0166), and C3bc/C3 ratio (HR 4.041 95% CI [1.673–9.758], *p* = 0.0019) were associated with 90-day mortality, while MASP-2, C4c, C3, and TCC were not. C4 had the highest ROC-AUC (0.748, [95% CI 0.649–0.847]), which was comparable to the AUC for SOFA score (0.753, [95% CI 0.649–0.857]), and SAPS II (0.862 [95% CI 0.795–0.929]).

**Conclusion:** In adjusted analyses, high admission levels of the C4c/C4 ratio, C3bc, and the C3bc/C3 ratio were significantly associated with a higher risk of death after 90 days while high admission levels of MASP-1 and C4 were associated with lower risk. In this cohort, these variables are better predictors of mortality in NSTI than C-reactive protein and Procalcitonin. C4's ability to predict mortality was comparable to the well-established scoring systems SAPS score II and SOFA on day 1.

## Introduction

Necrotizing soft-tissue infections (NSTIs) are infections with a necrotizing component involving any or all layers of the soft tissue compartment (1). They are rapidly progressive and can lead to sepsis, multisystem organ failure, and in 8–49% death (2). Surgical removal of all necrotic tissue is essential for survival and time to surgery an important determinant for the outcome (3–6). Yet it is common for patients to have additional areas of necrosis, and therefore require multiple operations with serial exploration and debridement until the infection is controlled (7). In 16% of patients, amputation is necessary (5).

If we had reliable tools to identify the patients requiring extensive surgery and discriminate them from the low-risk patients, we could improve survival rates, and avoid the extensive surgery in low-risk patients that currently leads to functional limitations in 30% of patients (8).

It is reasonable to look for such biomarkers in the complement system. Complement plays a crucial role in the microbial defense by mediating opsonization, sequestration, and lysis of pathogens (9), bridges the innate and adaptive system (10), and is activated by apoptotic and necrotic tissue (11). Uncontrolled complement activation is part of the hyperinflammatory response seen in sepsis and sepsis-related coagulopathy (12).

The complement system is divided into three converging proteolytic cascades; the classical, the lectin, and the alternative pathways. We have previously investigated the use of the lectin pathway initiator molecules and kinetic complement analyses as prognostic markers in NSTI patients (13), suggesting that pattern recognition molecules of the lectin pathway play an important role in these patients. Here, we further investigate the levels the lectin pathway associated enzymes MASP-1, MASP-2, MASP-3, the down-stream components C4, C3, the complement activation products C4c, C3bc, and the fluid phase analog of the terminal C5b-9 complement complex (TCC) in NSTI patients and a group of non-infected control patients.

## Materials and Methods

### Study Design and Setting

This prospective, observational study was conducted from February 2013 to March 2015 at the Copenhagen University Hospital (Rigshospitalet) where the national treatment of NSTI has been centralized. This is a substudy of the European INFECT project (ClinicalTrials.gov Identifier: NCT01790698).

Patients were screened at arrival to Rigshospitalet. Inclusion criteria were: (1) NSTI based on surgical findings with necrosis engaging any layers of the soft tissue compartments and (2) age ≥18 years. Patients were excluded if the NSTI diagnosis could not be confirmed during surgery.

Control patients were eligible for inclusion if they were: (1) undergoing elective orthopedic surgery at Rigshospitalet; and (2) were aged ≥18 years. Patients with ongoing infection or inflammatory conditions were excluded.

### Data Collection

We obtained clinical data from electronic records on age, sex, body mass index, chronic disease (diabetes, liver cirrhosis, chronic kidney disease, cardiovascular disease, chronic obstructive, pulmonary disease, peripheral vascular disease, immune deficiency, malignancy, rheumatoid disease), primary site of infection, microorganism, biochemistry, treatment, and the intensive care unit scoring systems; Simplified Acute Physiology Score II (SAPS II) and Sequential Organ Failure Assessment (SOFA) score day 1. SOFA score was altered as the Glasgow Coma Scale score was omitted. Vital status and time of death, if relevant, were extracted from the hospital database linked to the Danish Civil Registration System. The decision to amputate was based on the surgeon's evaluation. No protocol for amputation was used.

Standard blood analyses including platelet count, creatinine, leucocyte count, and C-reactive protein (CRP) levels were performed at the Department of Clinical Biochemistry, Rigshospitalet, as part of routine analyses, whereas sodium, potassium, hemoglobin, lactate, pH, base excess, pO_2_, and pCO_2_ were measured using an ABL 725 (Radiometer, Copenhagen, Denmark).

Upon admission, blood was drawn from an arterial line or central venous catheter into 9-mL vacuum tubes containing EDTA. For the control group, venous blood samples were drawn preoperatively. The samples were immediately put on ice until plasma was seperated from whole blood by centrifugation (within 40 min) at 2,400 G for 10 min and subsequently stored at −80°C.

MASPs, C4c, C3bc, and TCC were measured in the samples by Enzyme-linked Immunosorbent assays (ELISAs). MASP-1 and MASP-2 plasma levels were measured using commercial ELISA kits (MASP-1: USCN Life Sciences; catalog#: SEB895Hu, MASP-2: HycultBiotech Catalog #: HK326-02), as recommended by the manufacturer. Plasma levels of MASP-3, C4c, C3bc, and TCC were measured using previously described in house sandwich ELISAs (14–17). Total plasma concentrations of C4 and C3 were measured using an automated turbidimetric protein analyzer according to the manufacturer's instructions (SPAPLUS®, the Binding Site group LDT, Birmingham, UK).

### Outcome Measures

Our primary analysis was the association between complement parameters measured upon admission, 90-day mortality, and disease severity. We compared patients with septic shock vs. patients without, amputated vs. non-amputated patients and survivors vs. non-survivors. Septic shock was defined according to the criteria of Bone et al. (18). Amputation was defined as any amputation of a limb or penis.

Secondarily, we dichotomized every complement parameter by its median and investigated for associations with 90-day mortality. Patients were followed from inclusion until death, loss to follow up or March 2015, whichever came first.

### Statistical Analyses

The significance level was set at 0.05. Kolmogorov-Smirnov and Shapiro-Wilk normality tests were performed for all variables. Due to non-parametric distribution, continuous data were reported as median (IQR) and compared by the Kruskal–Wallis test. For *ad-hoc* pairwise analyses, we used Dunn's test. For categorical data, we reported absolute numbers, with proportions in parentheses, and used Pearson's chi-square test or Fisher's exact test for comparisons.

The prognostic value of the complement parameters for 90-day-mortality was investigated using Cox regression. For multivariate analysis, the first model was adjusted for sex and SAPS II, the second for age, sex, chronic disease (yes/no), and SAPS II. Data from the Cox analyses were presented as hazard ratios (HR) with 95 % confidence intervals (95% CI). The proportional hazard assumption was met for all parameters in our regression models. We were unable to calculate SAPS II in five patients due to missing data. These patients were excluded from the multivariate analysis. Receiver operating characteristic (ROC) curves with area under the curve (AUC) were reported for the complement parameters for 90-day mortality. Nine patients were excluded from the ROC analyses due to missing SAPS II, pH, base excess, or lactate. Statistical analyses were performed using SAS 9.4 (SAS Institute Inc., Cary, NC).

## Results

**Figure 1:**
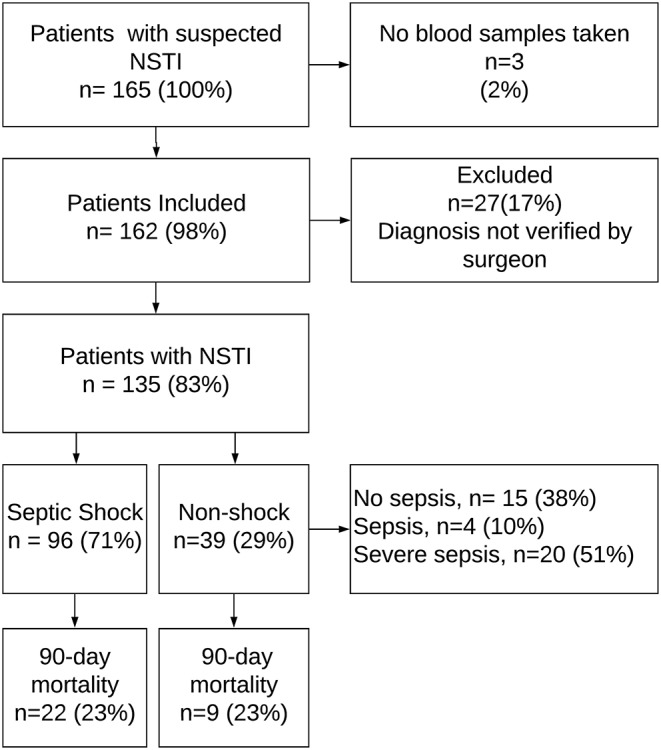
Flowchart of patient inclusion. NSTI, necrotizing soft-tissue infection. The figure is adapted from Figure 1 in Hansen et al. (19).

We included 135 NSTI patients (Figure 1) and 65 control patients, matched for age and sex. Ninety-six patients (71%) had septic shock, amputation was undertaken in 27 cases (20%), and 31 patients (23%) died within 90 days. Table 1 displays differences in the clinical data between surviving and non-surviving patients.

**Table 1:** Clinical data in survivors and non-surviving necrotizing soft-tissue infection patients.

**Variable**	**Total NSTI patients (*N* = 135)**	**Survivors (*N* = 104)**	**Non-survivors (*N* = 31)**	***p*-value**
Age, years	61.0 [52.0–69.0]	61.0 [50.0–67.5]	67.0 [58.0–70.0]	0.0693
Sex, male	84 (62.2)	63 (60.6)	21 (67.7)	0.4702
Body mass index*, kg/m*^2^^a^	26.1 [23.5–31.1]	25.6 [23.4–30.9]	27.8 [24.8–33.6]	0.0660
Active smoker	40 (29.6)	34 (32.7)	6 (19.4)	0.3023
High alcohol consumption^a^^b^	19 (19.6)	17 (22.4)	2 (9.5)	0.1892
Chronic disease	86 (63.7)	63 (60.6)	23 (74.2)	0.1664
Cardiovascular disease	58 (43.0)	41 (39.4)	17 (54.8)	0.1281
Chronic obstructive pulmonary disease	14 (10.4)	9 (8.7)	5 (16.1)	0.2308
Chronic kidney disease	12 (8.9)	6 (5.8)	6 (19.4)	0.0197
Diabetes	30 (22.2)	20 (19.2)	10 (32.3)	0.1257
Immune deficiency/AIDS	3 (2.2)	3 (2.9)	0 (0.0)	1.0000F
Liver cirrhosis	5 (3.7)	3 (2.9)	2 (6.5)	0.3236F
Malignancy	15 (11.1)	12 (11.5)	3 (9.7)	0.7723
Peripheral vascular disease	20 (14.8)	13 (12.5)	7 (22.6)	0.1655
Rheumatoid disease	10 (7.4)	8 (7.7)	2 (6.5)	0.8169
Hemoglobin, *mmol/L, lowest value*	2.2 [1.2–4.5]	1.9 [1.2–3.2]	4.3 [1.6–14.5]	0.0017
Na^+^, *mmol/L, lowest value*	5.7 [4.9–6.5]	5.8 [5.0–6.6]	5.2 [4.3–6.1]	0.0216
K^+^, *mmol/L, lowest value*	136.0 [132.0–138.0]	136.0 [132.0–138.0]	135.0 [131.0–139.0]	0.9979
Glucose, *mmol/L, highest value*	4.3 [4.0–4.9]	4.3 [4.0–4.6]	4.8 [3.8–5.5]	0.0617
Creatinine μmol/l, *highest value*^a^	8.3 [7.1–11.5]	8.3 [7.1–11.5]	8.2 [6.7–14.5]	0.8856
pO_2_*, kPa, from lowest PaO_2_/FiO_2_ ratio*^a^	119.0 [77.0–205.0]	101.0 [72.0–166.0]	216.0 [128.0–262.0]	0.0003
pH, *lowest value*	13.7 [10.9–19.6]	13.2 [10.9–18.6]	14.0 [9.4–22.3]	0.8351
Base excess, *mmol/L, lowest value*^a^	7.3 [7.2–7.4]	7.3 [7.2–7.4]	7.2 [7.1–7.3]	0.0014
Lactate, *mmol/L, highest value*	−5.5 [−9.4–−1.8]	−4.8 [−7.7–−1.6]	−10.2 [−13.9–−5.1]	0.0003
Steroid treatment^a^	16 (11.9)	9 (8.7)	7 (23.3)	0.0825F
Immunosupressing drugs^a^	12 (9.0)	10 (9.6)	2 (6.7)	1.0000F
Ventilator treatment	122 (90.4)	91 (87.5)	31 (100.0)	0.0384F
Renal replacement therapy	34 (25.2)	18 (17.3)	16 (51.6)	0.0001
Amputation	27 (20.0)	18 (17.3)	9 (29.0)	0.1520
Septic shock^c^	96 (71.1)	74 (71.2)	22 (71.0)	0.9840
SAPS II^a^	45.0 [35.0–52.0]	40.5 [32.0–48.5]	59.0 [48.0–76.0]	<0.0001
SOFA score day 1^a^	7.0 [4.0–9.0]	7.0 [4.0–9.0]	10.0 [8.0–12.0]	<0.0001
Primary site of infection				
Head/neck	21 (15.6)	16 (15.4)	5 (16.1)	0.9200
Chest	5 (3.7)	3 (2.9)	2 (6.5)	0.3236F
Abdomen	11 (8.1)	8 (7.7)	3 (9.7)	0.7229
Genitals/Perineum	36 (26.7)	31 (29.8)	5 (16.1)	0.1306
Upper extremity	15 (11.1)	14 (13.5)	1 (3.2)	0.1115
Lower extremity	47 (34.8)	32 (30.8)	15 (48.4)	0.0707
Microbiology				
Positive tissue or blood cultures	102 (75.6)	78 (75.0)	24 (77.4)	0.7832
Polymicrobial infection^a^	57 (55.9)	43 (55.1)	14 (58.3)	0.7821
*Staphylococcus aureus*^a^	17 (16.7)	13 (16.7)	4 (16.7)	1.0000F
Group A Streptococcus^a^	36 (35.3)	32 (41.0)	4 (16.7)	0.0290
Gram negative rods^a^	27 (26.5)	18 (23.1)	9 (37.5)	0.1613
Obligate anaerobes^a^	32 (31.4)	24 (30.8)	8 (33.3)	0.8129
Other bacteria^a^	21 (20.6)	15 (19.2)	6 (25.0)	0.5410F

*Values presented as Median [IQR] or N (column %) with Fisher's Exact-test (F) or Pearson's chi-square test*.

a *Data not available for all subjects. Missing values: Body mass index (n = 5): High alcohol consumption (n = 38), creatinine (n = 4), pO_2_ (n = 10), pH (n = 5), base excess (n = 5), lactate (n = 5), SOFA score (n = 6), steroid treatment (n = 1), Immunosuppresing drugs (n = 1), SAPS II (n = 5), SOFA score day 1 (n = 6), and positive tissue or blood cultures (n = 33)*.

b *High alcohol consumption: >14 units of alcohol/week (women); >21 units of alcohol/week (men)*.

c *Septic shock as defined by sepsis criteria by Bone et al. (18)*.

*SAPS II, Simplified Acute Physiology Score II; SOFA, Sequential Organ Failure Assessment; NSTI, Necrotizing soft tissue infection*.

### Complement Levels and NSTI Severity

MASP-1, C4, C4c, C3, decreased gradually, and C3bc/C3 levels increased gradually from controls to survivors, and from survivors to non-survivors, with significant differences both overall (Kruskal Wallis, *p* < 0.0003, *p* < 0.0001, *p* < 0.0001, *p* < 0.0001, and *p* < 0.0001, respectively), and between all groups as revealed by the *ad-hoc* test (Table 2). MASP-2 levels differed significantly between the groups (Kruskal Wallis, *p* = 0.0025). The *ad-hoc* analysis revealed that MASP-2 was significantly lower in non-survivors compared to survivors and controls but did not differ between controls and survivors. MASP-3 and TCC differed significantly between the groups (Kruskal Wallis, *p* < 0.0001). The *ad-hoc* test showed significantly lower MASP-3 and higher TCC levels in surviving and non-surviving patients when compared to controls resulting in an overall difference between the groups (Kruskal Wallis, *p* < 0.0001).

**Table 2:** Complement, leukocytes, and albumin in controls, surviving and non-surviving necrotizing soft-tissue infection patients.

**Variable**	**Total (*N* = 135)**	**Controls (*N* = 65)**	**NSTI survivors (*N* = 104)**	**NSTI non-survivors (*N* = 31)**	***p*-value**
MASP-1, *AU/L*	7.6 [6.5–8.9]	8.7 [7.5–10.2]^a^^b^	7.8 [6.8–9.0]^c^^b^	7.0 [5.8–8.0]^c^^a^	0.0003
MASP-2, *AU/L*	793.4 [504.1–1121.0]	977.5 [623.3–1464.3]^b^	874.5 [568.8–1182.5]^b^	551.9 [404.6–849.4]^c^^a^	0.0025
MASP-3, *AU/L*	65.4 [47.5–100.5]	109.3 [87.9–130.1]^a^^b^	64.3 [46.5–100.1]^c^	67.6 [56.6–104.8]^c^	<0.0001
C4, *g/L*^d^	0.17 [0.14–0.24]	0.24 [0.20–0.31]^a^^b^	0.19 [0.15–0.26]^c^^b^	0.13 [0.11–0.17]^c^^a^	<0.0001
C4c *AU/L*	134.6 [87.3–199.0]	194.7 [130.9–326.5]^a^^b^	150.4 [96.2–210.3]^c^^b^	99.6 [69.8–158.9]^c^^a^	<0.0001
C4c/C4^d^	752.9 [507.1–1165.8]	855.3 [569.8–1391.0]	720.2 [496.5–1163.0]	791.5 [571.3–1250.6]	0.2793
C3, *g/L*^d^	0.93 [0.75–1.2]	1.2 [1.1–1.4]^a^^b^	0.96 [0.79–1.2]^c^^b^	0.79 [0.56–0.98]^c^^a^	<0.0001
C3bc, *AU/L*	15.7 [9.2–24.2]14.7 [9.1–22.0]	12.5 [9.1–18.4]	15.2 [9.0–22.0]	17.7 [9.5–26.4]	0.1091
C3bc/C3^d^	16.5 [10.3–27.0]	10.7 [7.7, 14.7]^a^^b^	14.9 [9.8, 21.7]^c^^b^	24.3 [16.5, 40.6]^c^^a^	*<0.0*001
TCC, *AU/L*	1.5 [1.00–2.6]	0.35 [0.26–0.46]^a^^b^	1.5 [0.97–2.6]^c^	1.5 [1.1–2.7]^c^	<0.0001
Leukocyte count*, × 10/l, highest value*	16.9 [10.4–23.9]	7.2 [6.3–9.3]^a^^b^	16.5 [11.1–23.1]^c^	18.3 [8.5–27.2]^c^	<0.0001
C-reactive protein, *mg/L*^d^	222.0 [141.0–298.0]	2.0 [1.00–4.0]^a^^b^	224.5 [169.0–306.5]^c^^b^	165.0 [83.0–259.0]^c^^a^	<0.0001
Albumin, *g/L*	20.0 [16.0–22.0]	39.0 [36.0–41.0]^a^^b^	20.0 [16.5–22.5]^c^	19.0 [15.0–21.0]^c^	<0.0001

*Values presented as Median [IQR] with Kruskal-Wallis test. A significance level of 0.0167 was used for pairwise ad-hoc comparisons*.

a *Significantly different from NSTI survivors*.

b *Significantly different from NSTI non-survivors*.

c *Significantly different from controls*.

d *Data not available for all subjects. Missing values: C4 (n = 4), C4c/C4 (n = 4) C3 (n = 4), C3bc/c3 (n=4), leukocyte count (n = 3), and CRP (n = 4)*.

*NSTI, necrotizing soft-tissue infection*.

Table 3 displays the differences in the complement parameters across control patients and NSTI patients with and without septic shock. The median MASP-1 and C4c levels differed significantly between the groups (Kruskal Wallis, *p* = 0.0017 and *p* < 0.0001), and the *ad-hoc* analyses revealed significantly lower levels in shock patients compared to controls. There was a significant difference in median MASP-2 level between controls, non-shock and shock patients (Kruskal Wallis, *p* = 0.0390), but with no differences in the pairwise *ad-hoc* analyses. The levels of MASP-3, C4, and C3bc/C3 differed significantly between the groups (Kruskal Wallis, *p* < 0.0001, *p* < 0.0001, and *p* = 0.0002, respectively), with *ad-hoc* analyses showing significantly lower levels in non-shock and shock patients compared with controls. The C3 levels decreased gradually, from controls to non-shock, and from non-shock to septic shock with significant differences both overall (Kruskal Wallis, *p* < 0.0001), and between all groups as revealed by the *ad-hoc* test. Similarly, the median TCC levels increased gradually from the control patients to non-shock and non-shock to shock patients with significant differences both overall (Kruskal Wallis, *p* < 0.0001), and between all groups as revealed by *ad-hoc* analyses.

**Table 3:** Complement, leukocytes, and albumin in controls and necrotizing soft-tissue infection patients with and without septic shock.

**Variable**	**Total (*N* = 135)**	**Controls (*N* = 65)**	**Non-shock (*N* = 39)**	**Septic shock (*N* = 96)**	***p*-value**
MASP-1, *AU/L*	7.6 [6.5–8.9]	8.7 [7.5–10.2]^a^	8.4 [6.8–9.3]	7.4 [6.3–8.7]^b^	0.0017
MASP-2, *AU/L*	793.4 [504.1–1121.0]	977.5 [623.3–1464.3]	905.1 [551.9–1422.5]	717.3 [487.6–1015.0]	0.0390
MASP-3, *AU/L*	65.4 [47.5–100.5]	109.3 [87.9–130.1]^c^^a^	65.4 [48.4–86.2]^b^	65.5 [46.5–113.7]^b^	<0.0001
C4, *g/L*^d^	0.17 [0.14–0.24]	0.24 [0.20–0.31]^c^^a^	0.17 [0.14–0.26]^b^	0.17 [0.13–0.24]^b^	<0.0001
C4c *AU/L*	134.6 [87.3–199.0]	194.7 [130.9–326.5]^a^	158.2 [106.1–261.4]	125.9 [80.7–188.8]^b^	<0.0001
C4c/C4^d^	752.9 [507.1–1165.8]	855.3 [569.8–1391.0]	958.6 [522.1–1442.3]	671.5 [506.0–1086.0]	0.0828
C3, *g/L*^d^	0.93 [0.75–1.2]	1.2 [1.1–1.4]^c^^a^	1.04 [0.82–1.4]^b^^a^	0.89 [0.73–1.08]^b^^c^	<0.0001
C3bc, *AU/L*	15.7 [9.2–24.2]14.7 [9.1–22.0]	12.5 [9.1–18.4]	17.0 [9.5–22.4]	15.6 [9.1–24.2]	0.2460
C3bc/C3^d^	16.5 [10.3–27.0]	10.7 [7.7,14.7]^c^^a^	15.2 [9.3,23.0]^b^	16.7 [10.4,27.3]^b^	0.0002
TCC *AU/L*	1.5 [1.00–2.6]	0.35 [0.26–0.46]^c^^a^	1.3 [0.68–1.8]^b^^a^	1.7 [1.1–2.8]^b^^c^	<0.0001
Leukocyte count, × 10/l, *highest value*	16.9 [10.4–23.9]	7.2 [6.3–9.3]^c^^a^	15.5 [9.7–22.7]^b^	17.6 [12.3–24.4]^b^	<0.0001
C-reactive protein, *mg/L*^d^	222.0 [141.0–298.0]	2.0 [1.00–4.0]^c^^a^	201.0 [134.0–275.0]^b^	222.0 [154.0–301.5]^b^	<0.0001
Albumin, *g/L*	20.0 [16.0–22.0]	39.0 [36.0–41.0]^c^^a^	21.0 [18.0–23.0]^b^	18.5 [15.0–22.0]^b^	<0.0001

*Values presented as Median [IQR] with Kruskal-Wallis test. A significance level of 0.0167 was used for pairwise ad-hoc comparisons. Septic shock defined by criteria of Bone et al. (18)*.

a Significantly different from Septic Shock.

b Significantly different from controls.

c Significantly different from non-shock.

d *Data not available for all subjects. Missing values: C4 (n = 4), C4c/C4 (n = 4) C3 (n = 4), C3bc/c3 (n = 4), leukocyte count (n = 3), and CRP (n = 4)*.

Admission levels of C4, C4c, and C3 were lower in patients who underwent amputation vs. non-amputated (*p* = 0.0081, 0.0066, 0.0464, respectively; Supplementary Table 1).

### Survival Analysis

#### Univariate Analysis

The univariate models revealed that an increase in age, SOFA score, and SAPS II were associated with a higher hazard of death before 90-days. Chronic disease and amputation were not associated with mortality (Supplementary Table 2). Regarding the complement parameters, significant associations with 90-day mortality were found for MASP-1 (HR 0.372, 95% CI [0.171–0.808], *p* = 0.0125), MASP-2 (HR 0.297, 95% CI [0.133–0.666], *p* = 0.0032), C4 (HR 0.215, 95% CI [0.088–0.525], *p* = 0.0007), C4c (HR 0.371, 95% CI [0.171–0.806], *p* = 0.0123), C3 (HR 0.434, 95% CI [0.204–0.921], *p* = 0.0297), and C3bc/C3 (HR 3.283, 95% CI [1.468–7.344], *p* = 0.0038; Table 4).

**Table 4:** Cox regression analyses for 90-day mortality.

		**Unadjusted**			**Adjusted for sex, SAPS II**			**Adjusted for age, sex, chronic disease, and SAPS II**		
**Variables grouped by their medians**		**HR**	**95% CI**	***P*-value**	**HR**	**95% CI**	***P*-value**	**HR**	**95% CI**	***P*-value**
MASP-1	≤7.63 *AU/L*	1.000			1.000			1.000		0.0251
	>7.63 *AU/L*	0.372	[0.171–0.808]	0.0125	0.378	[0.164–0.872]	0.0226	0.368	[0.153–0.882]	
MASP-2	≤793.36 *AU/L*	1.000			1.000			1.000		0.0936
	>793.36 *AU/L*	0.297	[0.133–0.666]	0.0032	0.475	[0.202–1.119]	0.0886	0.473	[0.197–1.135]	
MASP-3	≤65.39 *AU/L*	1.000			1.000			1.000		0.3232
	>65.39 *AU/L*	1.133	[0.560–2.291]	0.7289	1.497	[0.710–3.157]	0.2896	1.466	[0.686–3.133]	
C4	≤0.17 *g/L*	1.000			1.000			1.000		0.0003
	>0.17 *g/L*	0.215	[0.088–0.525]	0.0007	0.162	[0.060–0.438]	0.0003	0.158	[0.057–0.434]	
C4c	≤134.63 *AU/L*	1.000			1.000			1.000		0.9072
	>134.63 *AU/L*	0.371	[0.171–0.806]	0.0123	1.033	[0.425–2.508]	0.9437	1.056	[0.424–2.631]	
C4c/C4	≤752.86	1.000			1.000			1.000		0.0250
	>752.86	1.473	[0.722–3.007]	0.2872	2.290	[1.078–4.867]	0.0312	2.543	[1.124–5.755]	
C3	≤0.93 *g/L*	1.000			1.000			1.000		0.9437
	>0.93 *g/L*	0.434	[0.204–0.921]	0.0297	1.005	[0.429–2.352]	0.9911	1.032	[0.433–2.460]	
C3bc	≤15.67 *AU/L*	1.000			1.000			1.000		0.0132
	>15.67 *AU/L*	1.657	[0.804–3.414]	0.1708	2.664	[1.195–5.938]	0.0166	2.841	[1.244–6.487]	
C3bc/C3	≤16.49	1.000			1.000			1.000		0.0017
	>16.49	3.283	[1.468–7.344]	0.0038	4.041	[1.673–9.758]	0.0019	4.301	[1.730–10.697]	
TCC	≤1.55 *AU/L*	1.000			1.000			1.000		0.1236
	>1.55 *AU/L*	0.961	[0.475–1.945]	0.9130	0.564	[0.267–1.192]	0.1338	0.554	[0.261–1.175]	

*Values are presented as Hazard ratios (HR) with 95% confidence intervals (95% CI) and p-values. Complement factors are divided according to median values with below median group as references. SAPS II, Simplified Acute Physiology Score II*.

### Multivariate Analysis

When adjusting the models for sex and SAPS II a level above the median MASP-1 (HR 0.378, CI 95% [0.164–0.872], *p* = 0.0226) and C4 (HR 0.162, 95% CI [0.060–0.438], *p* = 0.0003) remained significantly associated with lower 90-day mortality (Table 4). Additionally, a level above the median C4c/C4 ratio (HR 2.290 95% CI [1.078–4.867], *p* = 0.0312), C3bc (HR 2.664 95% CI [1.195–5.938], *p* = 0.0166), and C3bc/C3 ratio (HR 4.041 95% CI [1.673–9.758], *p* = 0.0019) were associated with an increased mortality. The remaining complement parameters were not associated with death in the adjusted models.

In a model where we adjusted for age, sex, chronic disease, and SAPS II, the same complement parameters yielded significant results (Table 4). Adding amputation as a covariate in our models did not change our results (data not shown).

### Receiver Operating Characteristics (ROC) Curves

We generated ROC curves for our explanatory variables predicting 90-day mortality and compared them to the ROC curves for SAPS II, SOFA score on day 1, and arterial blood gas values.

**Figure 2:**
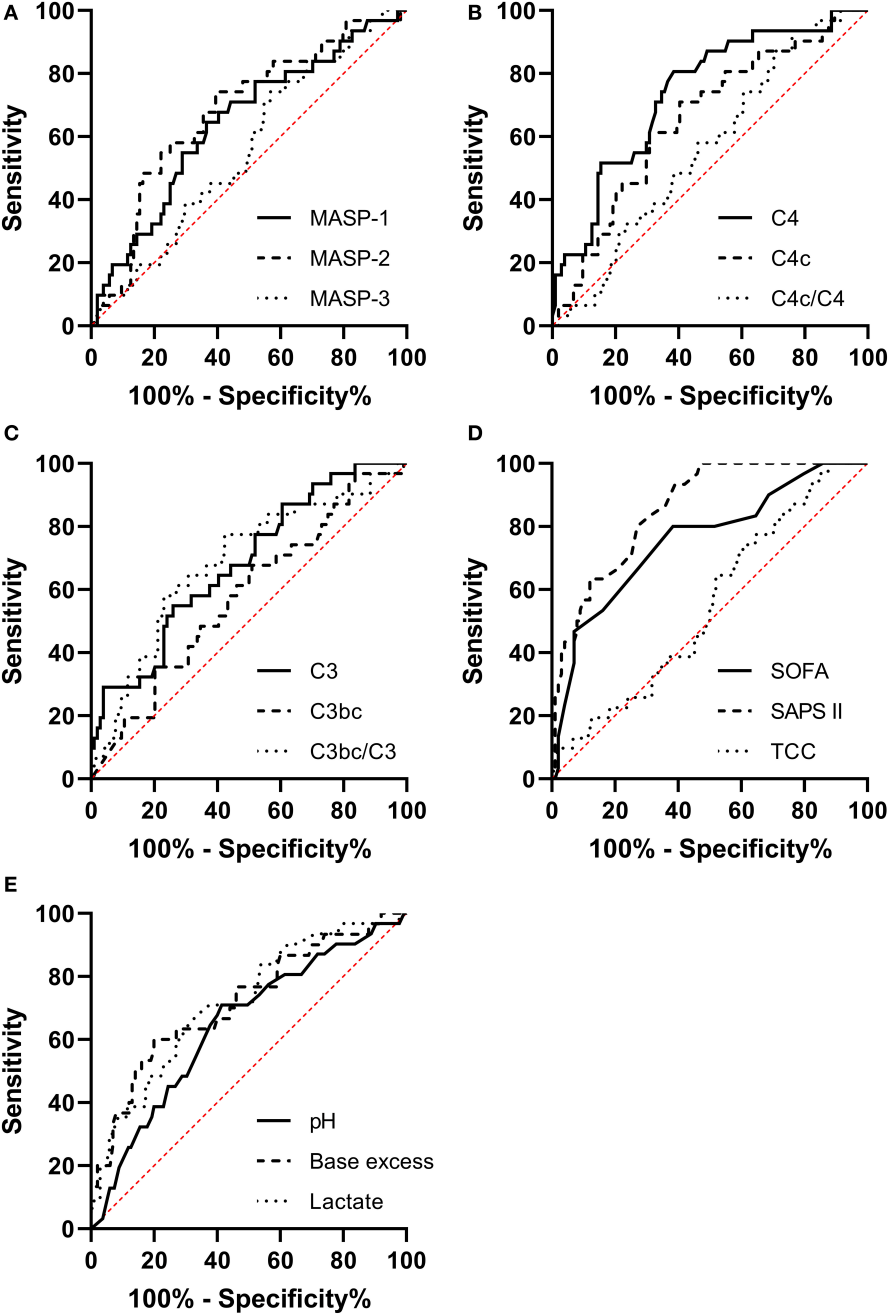
Receiver operating characteristic curves for predicting the 90-day mortality in necrotizing soft-tissue infection patients for **(A)** MASP-1, MASP-2, and MASP-3. **(B)** C4, C4c, and C4c/C4. **(C)** C3, C3bc, and C3bc/C3. **(D)** Sequential Organ Failure Assessment and score day 1 (SOFA), Simplified Acute Physiology Score II (SAPS II), and the terminal complement complex (TCC). **(E)** Arterial pH, base excess, and lactate.

The ROC Area Under the Curve (AUC) for MASP-1 (AUC 0.657, [95% CI 0.544–0.770]), MASP-2 (AUC 0.683, [95% CI 0.573–0.793]), C4 (AUC 0.748, [95% CI 0.649–0.847]), C4c (AUC 0.639, [95% CI 0.526–0.751]), C3 (AUC 0.686, [95% CI 0.577–0.794]), and C3bc/C3 (AUC 0.683, [95% CI 0.570–0.796]) were significantly different from random chance (AUC 0.5) as illustrated in Figure 2.

The SOFA score had an AUC of 0.753, [95% CI 0.649–0.857], SAPS II had an AUC of 0.862, [95% CI 0.795–0.929]. The AUCs for the complement parameters combined with SOFA score or SAPS II were not significantly different from the AUC of SOFA or SAPS II alone (data not shown).

For pH the AUC was 0.704 [95% CI 0.589–0.818], for base excess the AUC was 0.716 [95% CI 0.603–0.829], and for lactate the AUC was 0.731 [95% CI 0.631–0.831]. The ROC curve for C4 combined with pH (AUC 0.777 [95% CI 0.674–0.880]) was significantly different than the ROC curve for pH alone (*p* = 0.0374). C4 combined with base excess or lactate was not better than base excess or lactate alone (data not shown).

### Microbiology

Out of 135 patients, we could determine a responsible pathogen in 102 cases (76%). Two or more bacteria were responsible for 57 (56%) of these patients. The most common pathogen was Group A Streptococcus (GAS) with 36 infected patients (35%) (Table 1), but only 22 (22%) patients were only infected with GAS.

## Discussion

In this prospective, observational study we have shown that there's a relationship between the degree of complement activation and NSTI severity. Specifically, the levels of MASP-1, C4, C4c, C3, were significantly lower and C3bc/C3 significantly higher in non-survivors, than in survivors, who again differed significantly from controls. Similarly, the septic shock patients had significantly lower levels of C3, and higher levels of soluble TCC in their plasma, than the non-septic patients, who also differed significantly from the controls.

Interestingly, C4 and its breakdown product C4c both decreased with increasing severity. We expected an inverse proportionality between C4 and C4c, as we saw for C3 and its breakdown product C3bc. A probable explanation for this is a shorter half-life for C4c, leading to a faster clearance of C4c than of C3bc.

Overall, our findings are in line with studies on septic patients showing low C4 and C3 in non-survivors (20–22), and low MASP-1 in patients with disseminated intravascular coagulation (DIC) due to septic shock (23). This underlines that consumption of complement is directly linked to the pathophysiology in the hyperinflammatory reaction in NSTI as well as sepsis in general.

We have previously examined several complement system associated pattern recognition molecules (PRMs) such as CRP, Pentraxin-3, MBL, ficolin-1, ficolin-2, and ficolin-3 as biomarkers for risk stratification in the same cohort of NSTI patients (13, 19). In the survival analysis, ficolin-2 was the most promising of the above-mentioned PRMs, but the association with mortality disappeared after adjusting for SAPS II (13). Biomarkers outside of the complement system such as Procalcitonin and soluble urokinase plasminogen activator receptor (suPAR) have also been examined and they were not associated with mortality in the adjusted analyses (19, 24).

Here, we adjusted for the same covariates as previously and found above-median admission levels of MASP-1 and C4 to be independently associated with a lower risk of death before 90-days, when compared to below or equal to median admission levels. Above median admission levels of the C4c/C4 ratio, C3bc, and the C3bc/C3 ratio were independently associated with increased risk of death before 90-days from admission when compared to below or equal to median admission levels. These findings clearly suggest that complement consumption predicts a poor outcome in NSTI and that the ability to maintain circulating complement is linked to survival.

There are several, non-exclusive theories explaining the biological mechanism behind complement consumption in hyperinflammatory states. One theory is that consumption is a result of complement deposition in the necrotic and infected tissue. In addition, the complement protein production is probably affected in multiple organ failure, and there could also be a dilution effect with fluid resuscitation, although fluid resuscitation cannot explain why TCC concentration is higher in shock or non-survivors.

We lacked statistical power to examine the pathogens in the NSTI patients, but the MASP-1 findings indicate that the lectin pathway is involved in the pathophysiology. This is also supported by our previous findings showing that the NSTI patients had significantly lower baseline MBL and ficolin levels than the non-infected controls (13), indicating that the lectin pathway PRM's are also consumed in NSTI. To better understand the role of the classical pathway in NSTI we will have to proceed with C1q measurements.

If the depleted complement in plasma is reflecting an overactivation and dysregulation of complement in the infected and necrotic tissue; complement inhibition could possibly be beneficial. Complement inhibition has already been shown to improve survival in a septic baboon model (25). We think our findings warrant further studies on the use of C1-inhibitor and C5 inhibition in animal models.

The well-established clinical SOFA score and SAPS II also predicted the risk of death in adjusted Cox analyses. Scoring systems are necessary to monitor the patients over several days, however, using scoring systems as admission triage can be problematic as the necessary variables are often available after at least 24 h of admission. At that point, many interventions have already been made (26). The new SAPS 3, can be calculated quicker since it uses data available within 1 h of ICU admission (27), but, as Polzik et al. points out; it requires more variables to calculate, many of which may be missing upon admission (24). We wanted to compare the ROC curves for our complement parameters to the ROC curves for SAPS II, SOFA score on day 1, and the quickly available pH, base excess and lactate levels.

The prognostic value of MASP-1, MASP-2, C4, C4c, C3, C3bc/C3, pH, base excess, and lactate were similar to the prognostic value of SOFA score day 1, but only the AUC for C4, base excess and lactate had 95% CI's that overlapped with the AUC for SAPS II (0.862, [95% CI 0.795–0.929]). C4 had a higher AUC (0.748, [95% CI 0.649–0.847]) than the arterial gas values. Additionally, the AUC for C4 and pH combined (AUC 0.777 [95% CI 0.674–0.880]) was significantly higher than the AUC for pH alone (0.704, [95% CI 0.589–0.818]), further underlining C4's potential as a prognostic marker.

To our knowledge, this is the first study to examine complement parameters in a large, prospective cohort of NSTI patients. The study includes all NSTI patients transferred to our tertiary care hospital during the study period, which raises external validity. Besides, there was no loss to follow up, and the investigators performing analyses were blinded. Nevertheless, it is pertinent to mention some limitations of our study. NSTI patients are a heterogeneous group, and the complement parameters could be influenced by unknown confounders, as in other observational studies. Secondly, some patients might have died, before transfer to our tertiary hospital center, increasing the risk of selection bias. Fifteen patients (11%), did not fulfill the sepsis criteria according to Bone et al. which could be a result of this selection bias but could also be due to the variable time course to fulminant disease (18). Our controls were sampled preoperatively, while the NSTI patient's primary debridement often happened before transfer to our hospital. This means that the surgery, in and of itself, could bias the differences we measure between controls and patients. In addition, the controls were all elective orthopedic surgery patients. An extremity is the primary affected site in most NSTI cases but ideally, we should also have included controls from other surgical specialties. Lastly, we did not account for varying levels of complement proteins throughout the study, although the baseline level is most important for initial risk stratification and triage.

Since MASP-1, C4, the C4c/C4 ratio, C3bc, and the C3bc/C3 ratio showed great potential as prognostic markers next steps could be verification in a randomized control trial investigate differences in mortality, where one study arm uses complement admission levels to identify high-risk patients and then investigate for differences in mortality between groups. It would also be interesting to examine whether complement levels can discriminate non-necrotizing soft-tissue patients from NSTI to elucidate whether the complement levels could play a role diagnostically.

## Conclusions

We have shown that there is a relationship between the degree of complement activation and NSTI severity suggesting that consumption of complement is directly linked to the pathophysiology in NSTI. Additionally, we have shown that low levels of MASP-1, C4 and high levels of the C4c/C4 ratio, C3bc and the C3bc/C3 ratio are associated with 90-day mortality in unadjusted and adjusted survival analyses, establishing these markers as better predictors of mortality than the currently used NSTI biomarkers C-reactive protein and Procalcitonin, at least in this cohort.

C4's ability to predict mortality was comparable to the well-established scoring systems SAPS score II and SOFA on day 1.

## Data Availability Statement

The raw data supporting the conclusions of this article will be made available by the authors, without undue reservation, to any qualified researcher.

## Ethics Statement

The studies involving human participants were reviewed and approved by the Regional Ethics Committee of the Capital Region of Denmark (H-2-2014-071) and the Danish Data Protection Agency (J. no. 30-1282) and registered at ClinicalTrials.gov (NCT02180906). The patients/participants or their legal guardians provided their written informed consent to participate in this study.

## Author Contributions

PG and OH conceived the study. PG, MH, and MM participated in study design and coordination. MH and MM participated in data acquisition and maintain the database for analysis. MH, KP, and MK analyzed the data. MK drafted the first manuscript. PG, OH, KP, CH, MH, MM, and MK contributed to critical revision of the work.

### Conflict of Interest

The authors declare that the research was conducted in the absence of any commercial or financial relationships that could be construed as a potential conflict of interest.

## Abbreviations

AUC: area under the curve
CI: confidence interval
CRP: C-reactive protein
ELISA: enzyme-linked immunosorbent assays
HR: hazard ratio
IQR: interquartile range
NSTI: necrotizing soft-tissue infection
P: *p*-value
PRMs: pattern recognition molecules
ROC: Receiver Operating Characteristics
SAPS II: Simplified Acute Physiology Score II
SOFA: Sequential Organ Failure Assessment
suPAR: soluble urokinase plasminogen activator receptor
TCC: terminal C5b-9 complement complex.
