# Supplementary material for: Complement Activation Is Associated With Mortality in Patients With Necrotizing Soft-Tissue Infections—A Prospective Observational Study
Source: Front Immunol. 2020 Jan 31;11:17. doi: 10.3389/fimmu.2020.00017 (PMC7006023; doi:10.3389/fimmu.2020.00017)
Supplement: Supplementary file 1 [file Table_1.docx]

| **Supplementary Table 1 -** **Complement, leukocytes, and albumin in non-amputated and amputated necrotizing soft-tissue infection patients.** | | | | |
| --- | --- | --- | --- | --- |
|  |  |  |  |  |
| Variable | Total (N=135) | Non-amputated (N=108) | Amputated (N=27) | p-value |
| MASP-1, *AU/L* | 7.6[6.5–8.9] | 7.6[6.6–8.9] | 8.0[6.1–9.0] | 0.7476 |
| MASP-2, *AU/L* | 793.4[504.1–1121.0] | 832.6[522.2–1157.2] | 620.1[463.5–944.0] | 0.1368 |
| MASP-3, *AU/L* | 65.4[47.5–100.5] | 68.0[47.0–98.4] | 62.5[49.3–119.2] | 0.9080 |
| C4, *g/L* | 0.17[0.14–0.24] | 0.18[0.14–0.25] | 0.15[0.08–0.19] | 0.0081 |
| C4c, *AU/L* | 134.6[87.3–199.0] | 150.4[96.5–209.1] | 97.9[67.5–140.1] | 0.0066 |
| C4c/C4 | 752.9[507.1–1165.8] | 737.7[527.0–1165.5] | 775.0[424.0–1273.4] | 0.8993 |
| C3, *g/L* | 0.93[0.75–1.2] | 0.94[0.79–1.2] | 0.78[0.59–1.08] | 0.0464 |
| C3bc, *AU/L* | 15.7[9.2–24.2] | 15.5[9.4–22.0] | 18.2[8.9–30.2] | 0.4760 |
| C3bc/C3 | 16.5[10.3–27.0] | 15.7[10.1–22.9] | 24.2[12.6–38.7] | 0.0703 |
| TCC, *AU/L* | 1.5[1.00–2.6] | 1.6[1.00–2.5] | 1.5[1.01–2.7] | 0.6819 |
| leukocyte count, *x 10/l, highest value* | 16.9[10.4–23.9] | 16.5[10.1–24.2] | 17.4[12.4–21.8] | 0.6264 |
| C-reactive protein, *mg/L* | 222.0[141.0–298.0] | 224.5[144.5–306.5] | 202.0[90.0–244.0] | 0.1228 |
| Albumin, *g/L* | 20.0[16.0–22.0] | 20.0[16.0–22.5] | 18.0[15.0–21.0] | 0.3774 |
| Values presented as Median [IQR] with Kruskal-Wallis test. | | | | |
