# Supplementary material for: Complement Activation Is Associated With Mortality in Patients With Necrotizing Soft-Tissue Infections—A Prospective Observational Study
Source: Front Immunol. 2020 Jan 31;11:17. doi: 10.3389/fimmu.2020.00017 (PMC7006023; doi:10.3389/fimmu.2020.00017)
Supplement: Supplementary file 2 [file Table_2.docx]

| **Supplementary Table 2 – Univariate Cox regressions for 90-day mortality** | | | |
| --- | --- | --- | --- |
| Variable | HR | 95% CI | P-value |
| Age | 1.042 | [1.012– 1.072] | 0.0055 |
| Sex, male | 1.418 | [0.698– 2.882] | 0.3344 |
| Chronic disease (y/n) | 1.804 | [0.848– 3.836] | 0.1255 |
| SOFA score day 1 | 1.210 | [1.100– 1.330] | <0.0001 |
| SAPS II | 1.065 | [1.046– 1.084] | <0.0001 |
| Amputation | 1.347 | [0.798–1.347] | 0.2646 |
| Values are presented as Hazard ratios (HR) with 95% confidence intervals (95% CI) and p-values. *SOFA* Sequential Organ Failure Assesment, *SAPS II* Simplified Acute Physiology Score II. | | | |
